# Developmental Exposure to a Mixture of Unconventional Oil and Gas Chemicals Increased Risk-Taking Behavior, Activity and Energy Expenditure in Aged Female Mice After a Metabolic Challenge

**DOI:** 10.3389/fendo.2019.00460

**Published:** 2019-07-25

**Authors:** Victoria D. Balise, Jennifer N. Cornelius-Green, Brittany Parmenter, Sierra Baxter, Christopher D. Kassotis, R. Scott Rector, John P. Thyfault, Silvia Paterlini, Paola Palanza, Daniel Ruiz, Robert Sargis, Susan C. Nagel

**Affiliations:** ^1^Department of Obstetrics, Gynecology and Women's Health, University of Missouri, Columbia, MO, United States; ^2^Department of Biological Sciences, University of Missouri, Columbia, MO, United States; ^3^Nicholas School of the Environment, Duke University, Durham, NC, United States; ^4^Department of Nutrition and Health Exercise Physiology, University of Missouri, Columbia, MO, United States; ^5^Division of Gastroenterology and Hepatology, School of Medicine, University of Missouri, Columbia, MO, United States; ^6^Research Service, Harry S. Truman Memorial Veterans Medical Center, Columbia, MO, United States; ^7^Department of Molecular and Integrative Physiology, University of Kansas Medical Center, Kansas City, KS, United States; ^8^Kansas City VA Medical Center, Research Service, Kansas City, MO, United States; ^9^Unit of Neuroscience, Department of Medicine and Surgery, University of Parma, Parma, Italy; ^10^Department of Medicine, University of Illinois at Chicago, Chicago, IL, United States

**Keywords:** unconventional oil and gas, energy expenditure, endocrine disrupting chemicals, activity, hydraulic fracturing, metabolic

## Abstract

Chemicals used in unconventional oil and gas (UOG) operations can act as endocrine disrupting chemicals and metabolic disruptors. Our lab has reported altered energy expenditure and activity in C57BL/6J mice that were preconceptionally, gestationally, and lactationally exposed via maternal drinking water to a laboratory-created mixture of 23 UOG chemicals from gestational day 1 to postnatal day 21 in 7-month-old female mice with no change in body composition. We hypothesized that allowing the mice to age and exposing them to a high fat, high sugar diet might reveal underlying changes in energy balance. To investigate whether aging and metabolic challenge would exacerbate this phenotype, these mice were aged to 12 months and given a high fat, high sugar diet (HFHSD) challenge. The short 3-day HFHSD challenge increased body weight and fasting blood glucose in all mice. Developmental exposure to the 23 UOG mixture was associated with increased activity and non-resting energy expenditure in the light cycle, increased exploratory behavior in the elevated plus maze test, and decreased sleep in 12 month female mice. Each of these effects was seen in the light cycle when mice are normally less active. Further studies are needed to better understand the behavioral changes observed after developmental exposure to UOG chemicals.

## Introduction

Endocrine-disrupting chemicals (EDCs) are chemicals capable of disrupting normal hormone action and can be found in food, consumer products, and our environment (1). EDCs have been linked to health problems including obesity, diabetes, reproduction, cancers, and neurodevelopmental problems (2). EDCs that can alter the predisposition to obesity and metabolic disease are now termed metabolic-disrupting chemicals (MDCs); these chemicals can promote metabolic changes that can result in type 2 diabetes, fatty liver, and/or obesity (3, 4).

We have previously reported that chemicals used in unconventional oil and gas (UOG) operations can act as EDCs. UOG operations use directional drilling and hydraulic fracturing to release natural gas and oil that were previously inaccessible via traditional drilling methods. While only a few dozen chemicals may be used in a single well in this process, over 1,000 different chemicals are used industrywide. We have previously shown that 23 commonly-utilized UOG chemicals tested could disrupt five nuclear hormone receptors [estrogen (ER), androgen (AR), progesterone (PR), glucocorticoid (GR), and thyroid (TR)] (5).

Wastewater from UOG processes can contaminate surface and ground water. UOG activities and processes, such as transportation, well casing failure, wastewater spills, and direct injection have been linked to drinking water contamination (6–10). We have reported that surface water downstream of an injection well disposal site had higher antagonistic activity for ER, AR, PR, GR, and TR compared to surface water upstream of the site (11).

Exposure to MDCs during susceptible windows of development may result in adverse health outcomes (12–14). In a systematic review by Elliot et al., 41 UOG chemicals were identified as being linked to developmental toxicity (15). We recently demonstrated that UOG chemicals and wastewater can act as metabolic disruptors, promoting lipid accumulation and stimulating adipocyte differentiation as well as promoting the proliferation of pre-adipocytes, via activation of peroxisome proliferator activated receptor gamma (PPARγ) or other mechanisms (16). We also found that female mice prenatally exposed to a mixture of these 23 UOG chemicals (which exhibited antagonistic activity for ER, AR, PR, GR, and TR) from gestation day 11 through 19 had altered organ weights, reproductive endpoints, and increased body weights at postnatal days 7, 13, and 21 (5). There is accumulating evidence that UOG chemicals and wastewater are associated with developmental programming of metabolism.

In a recent study, we extended this line of research to encompass a longer exposure window from gestation day 1 to postnatal day 21. Interestingly, we found an opposite effect on female body weight with the longer developmental exposure: female mice exposed to the 23 UOG chemical mixture weighed less on postnatal day 7 and 21, as opposed to mice exposed from PND 11-18, who weighed more than vehicle controls (17). These female offspring were followed into adulthood, and showed no changes in body composition (body weight, fat mass, or lean mass) at 7 months of age, but exhibited a decrease in resting energy expenditure and activity in the dark cycle compared to vehicle-treated controls (17).

Developmental exposure to metabolic disrupting chemicals can be subtle and an absence of altered body composition at one adult age does not necessarily mean an absence of altered metabolism throughout adulthood (18). The endocrine system maintains homeostasis and utilizes many compensatory mechanisms, such as feedback loops. To discern underlying developmental programming, a “second hit” is often needed to challenge the system beyond compensation (3). Common challenges include stressors such as changes in activity, light exposure, or diet. A high fat, high sugar diet (HFHSD) is often-employed as a metabolic challenge to induce obesity or metabolic dysfunction. Since we previously observed altered resting energy expenditure in female mice exposed to the 23 UOG mixture at 7 months of age, but no change in body composition, we hypothesized that allowing the mice to age and exposing them to a HFHSD might reveal underlying metabolic disruption.

## Materials and Methods

### Animals

This study was carried out in accordance with the recommendations National Research Council's Guide for the Care and Use of Laboratory Animals. The protocol was approved by the University of Missouri Animal Care and Use Committee.

### Chemical Mixture and Treatment

Female C57BL/6J mice (purchased from Jackson Laboratories) were mated at 9 months of age. It should be noted that these dams were used in a previous study by our lab, in which they were also mated and exposed to the same mixture of 23 UOG chemicals used in the present study. Offspring outcomes from the first experiment were reported previously (5, 19). These females received the same concentrations of chemical mixtures that were randomly assigned in the previous study (5). Dams (*n* = 14, 9, 11, 8, and 10) were exposed to the chemical mixture at concentrations of 0, 0.01, 0.10, 1.0, or 10 μg/mL, respectively, for 5 weeks prior to mating. Chemical exposure was paused while females were mated in order to bypass the window of fertilization, and to avoid consumption of treatment chemicals by the males. Treatment was resumed at gestational day one (1 day after presence of copulatory plug) and continued through weaning of the F1 generation at PND 21. As reported in Balise et al. (17), dams had no differences in body weight or water consumption (Supplementary Figure 1). Also, there were no differences reported in either litter size or sex ratio (Supplementary Figure 1) (17).

From birth to 6 months of age, F1 mice were housed in a barrier facility where both feed (LabDiet 5053: 13% kcal fat, 3.25% kcal sucrose) and acidified water were sterilized and provided *ad libitum*. Glass bottles and polysulfone cages were used for all animals. After the F1 generation reached 6 months of age, they were transferred to a conventional facility where the metabolic instrumentation was located. In this facility, they received non-sterilized feed (LabDiet 5053: 13% kcal fat, 3.25% kcal sucrose) and non-acidified, non-sterilized water. Both facilities were temperature controlled and kept on a 12 h light/dark cycle.

The 23 chemicals were mixed equimass in 200 proof ethanol and added to drinking water such that each individual chemical was present at a concentration of 0.01, 0.10, 1.0, or 10 μg/mL in a 0.2% ethanol vehicle in acidified drinking water. Water bottles were changed twice per week to ensure consistent chemical concentrations throughout the dosing period. Water consumption was calculated as the difference in the weight of the water bottle before and after use every time the bottle was changed. Dosages based on weight of the dam and the amount of water consumed were calculated as 1.5, 15, 150, and 1,500 μg/kg/day. Offspring in litters that had less than one male or female per litter were excluded from analysis. After application of inclusion criteria, 0, 1.5, 15, 150, and 1,500 μg/kg/day treatment groups included *n* = 9, 11, 9, 10, and 10 total individual animals, and *n* = 6, 4, 5, 4, and 4 pups from unique litters, respectively (Supplementary Table 1).

### Elevated Plus Maze Behavioral Test

An elevated plus maze (EPM) test was conducted at 11 months of age. Tests were started in the light cycle, 3 h before the initiation of the dark cycle. Mice were placed gently into the maze facing an open arm and were video-recorded for a period of 5 minutes. Time spent in open arms, closed arms, and center were reported as a percentage of the five-min recording (20).

### Time Line of High Fat and High Sugar Diet (HFHSD) Challenge and Necropsy

**Figure 1:**
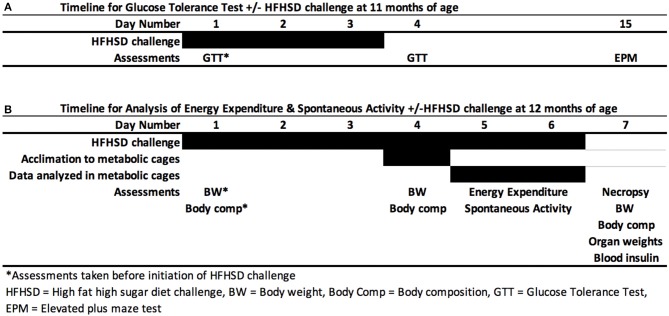
Metabolic challenge. Female mice were given a high fat high sugar diet (HFHSD) at 11 months **(A)** and 12 months of age **(B)**. After 3 days, either a glucose tolerance test (GTT) was performed at 11 months of age **(A)** or mice were placed in metabolic cages for indirect calorimetry for 3 days **(B)**. Mice were allowed to acclimate to the new cage environment on first day (Day 4) of indirect calorimetry and Days 5 and 6 were used for data analysis.

Mice were given two high fat, high sugar diet (HFHSD) challenges. Two separate challenges were needed since GTT could not be performed while mice were in metabolic cages (Figure 1). F1 females were given a HFHSD for 6 days. The high fat, high sugar diet (HFHSD) metabolic challenge contained 45% kcal fat and 17% kcal sucrose; Research Diet D12451). The first HFHSD challenge was given at 11 months of age to perform glucose tolerance tests (GTT). GTT was assessed before and 3 days after the HFHSD challenge (Figure 1A). The second HFHSD challenge was given at 12 months of age, to assess energy expenditure and activity in the metabolic cages (Figure 1B). Mice were given the HFHSD challenge for 3 days and placed into indirect calorimetry cages on Day 4 for 3 additional days, and necropsy was performed on day 7. Necropsy was performed by carbon dioxide asphyxiation followed by cervical dislocation.

### Indirect Calorimetry

Energy expenditure, activity, food and water intake, and behavior were assessed via indirect calorimetry using the Promethion from Sable Systems Int., (Las Vegas, NV). Mice were individually housed in the cages for 72 h. The first 24 h were used as an acclimation period, and the last 48 h were used for analysis. The 12 h light cycles and 12 h dark cycles were separately analyzed. Outcomes were calculated with macros provided by the manufacturer (21).

Energy expenditure was obtained from measured oxygen consumption. Total energy expenditure was calculated using the Weir equation in kilocalories and was the overall energy expenditure for a 12 h cycle. Resting energy expenditure was extrapolated from the lowest average energy expenditure in a 30 min window within a 12 h cycle. Non-resting energy expenditure was calculated for each 12 h cycle by subtracting resting energy expenditure from total energy expenditure. Active energy expenditure was extrapolated from the highest average energy expenditure for 15 min within a 12 h cycle.

Activity and meters traveled were measured by infrared beams that track movement in horizontal (X and Y plane) and vertical directions (Z plane). Spontaneous activity was activity in the X, Y, and Z direction, ambulatory activity in the X and Y directions and rearing activity in the Z direction. Meters traveled counted all meters in the X, Y, and Z direction, while pedestrian meters measured meters in the X and Y directions only. Food consumption and food bouts (number of times the animal made contact with the food hopper) were also measured.

### Body Composition

At 12 months of age, body weight, fat mass, and lean mass were measured just prior to starting the HFHSD metabolic challenge on Day 1, and on Day 4 and 7. Fat and lean mass were measured using the EchoMRI-900 (EchoMRI, Houston, TX) (17). Fat and lean percentages were calculated by dividing fat or lean mass by total body weight.

### Glucose Tolerance Test

Glucose tolerance tests were performed in mice at 11 months of age before and after HFHSD on Day 0 and Day 4, respectively. Sixteen mice from all treatment groups were randomized to be tested per day over the course of 1 week. Mice were weighed at 1000 h and fasted from 1000 to 1600 h. A baseline (0 min) blood sample was collected via tail snip at 1600-1630 hrs, and blood glucose was determined using a glucose monitor (Accu-Chek Aviva Plus). Immediately after the baseline measurement was taken, 250 mg/mL glucose was injected intraperitoneally at 1 gram of glucose per kg of lean mass (22, 23). Blood glucose concentrations were measured at 30, 60, and 120 min after injection (24).

### Liver Triacylglycerol Concentration

At necropsy, livers were collected and stored at −80°C. Liver triacylglycerol concentration was determined as previously described using a commercially available kit (F6428, Sigma, St. Louis, MO) (25).

### Serum Insulin Concentration

Blood was collected at time of necropsy by cardiac puncture and stored on ice. Centrifugation was performed to separate serum, and samples were stored at −80°C. Serum insulin concentrations were measured using a commercially available mouse insulin ELISA kit according to manufacturer's instructions (Alpco Diagnostics, cataolog #80-INSMSU-E01, Salem, MA).

### Pancreas Analysis

#### Pancreatic Histology and Immunohistochemistry (IHC)

At the time of necropsy, pancreata were dissected and fixed in 4% paraformaldehyde overnight and paraffin-embedded. Tissue sections (5 μm in thickness) were immunostained with the following primary antibodies (all at 1:500 dilution): polyclonal guinea pig anti-porcine insulin (DAKO, Carpinteria, CA), mouse monoclonal anti-human glucagon (Sigma-Aldrich, St. Louis, MO), polyclonal goat anti-somatostatin (Santa Cruz, Santa Cruz, CA), and DAPI (Invitrogen, Carlsbad, CA). The primary antibodies were detected using a combination of DyLight 488, 549, and 649-conjugated secondary antibodies (1:200, Jackson Immuno Research Laboratory, West Grove, PA).

#### Image Capture and Islet Quantification

As previously described (26, 27), microscopic images of pancreatic sections were taken with an Olympus IX8 DSU spinning disk confocal microscope (Melville, NY) with Stereo Investigator imaging software (SI, Micro Bright Field, Williston, VT). A modified method of “virtual slice capture” was utilized. Quantification of cellular composition (i.e., each area of β-, α-, and δ-cell populations, and islet area by automated contouring of each islet) was carried out using custom-written scripts for Fiji/ImageJ (http://rsbweb.nih.gov/ij/). MATLAB (MathWorks, Natick, MA) was used for mathematical analyses (26, 27).

### Vaginal Cytology and Estrus Cycle Analysis

Vaginal smears were taken daily for 14 days between 9 and 10 months of age. The vaginal cells of each female were washed out the day before smears were performed and collected in a 96 well plate. Vaginal cytology was assessed and estrus cycle stage given based on proportion of cell types, as previously reported (28).

Mice were in metabolic cages for 3 days immediately preceding necropsy, so estrus cyclicity could not be monitored as mice could not be removed from the metabolic cages. Vaginal cytology was assessed on the day of necropsy. The first single smear contains a mixture of the preceding days vaginal cells. On the day of necropsy, smears contained only cornified epithelium and did not contain white blood cells and were thus concluded to be in persistent estrus at 12 months of age.

### Statistics

Data were analyzed with a linear mixed model using SPSS version 32. This model was selected so that litter could be incorporated as a random effect. Treatment group and dates of measurement were included as fixed effects for body weight, fat mass, lean mass, fat percent, lean percent, food consumption, and activity. For analyses of body weight, litter size was included as a fixed effect. For analysis of energy expenditure, body weight was included as a fixed effect.

Data that were not normally distributed were transformed to achieve normality. Results are displayed in all figures as the estimated marginal means, back transformed for presentation if transformation was necessary. Differences between vehicle and treatment groups were analyzed using Fisher's Least Significant Difference tests with 95% confidence intervals. Elevated plus maze decision making was analyzed using Fisher's exact test. All treatment groups were compared to vehicle only.

## Results

### Exploratory Behavior

**Figure 2:**
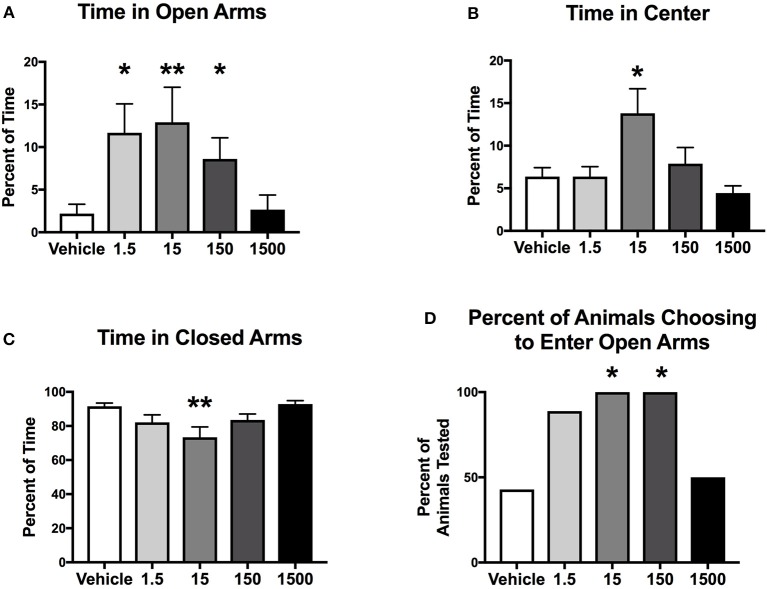
Exploratory activity of female mice at 11 months of age. Estimated marginal means (±SEM) of exploratory activity 3 h before initiation of the dark cycle. Percentage of time spent in the elevated plus maze in open arms **(A)**, center of the maze **(B)**, and closed arms **(C)**. Percent of animals within treatment group choosing to enter open arms **(D)** (*n* = 7, 9, 7, 6, 6, respectively, for vehicle, 1.5, 15, 150, and 1500 μg/kg/day treatment groups). ^*^p < 0.05 and ^**^p < 0.0125 relative to vehicle. Model included covariates: litter and assessment date.

Mice were introduced to an elevated plus maze for 5 min at the end of the light cycle at 11 months of age (Figure 1). Time spent in center, open and closed arms was measured to assess exploratory behavior. Exploratory activity (defined as the amount of time spent in open areas of the maze) was altered by developmental exposure to the UOG chemical mixture. Animals spent 400, 490, 290% more time in the open arms in the 1.5, 15, and 150 μg/kg/day groups, respectively, relative to vehicle control (Figure 2A). Treatment 150 μg/kg/day also spent 115% more time in the center and 20% less time in closed arms relative to vehicle (Figures 2B,C). Additionally, 100% of mice in the 15 and 150 μg/kg/day groups chose to enter open arms vs. 43% in the vehicle control group (Figure 2D).

### Response to the High Fat High Sugar Diet (HFHSD)

**Figure 3:**
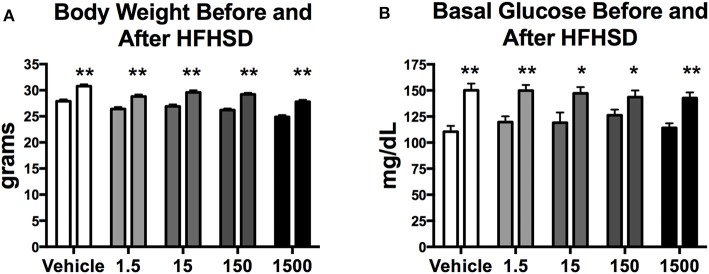
Body weight and basal glucose levels before and after high fat high sugar diet (HFHSD) at 11 months of age. Estimated marginal means (±SEM) of body weight before and after 3 days on HFHSD **(A)**, and basal blood glucose levels before and after 3 days on HFHSD **(B)**. For body weight *n* = 10, 8, 9, 10, 9, respectively, for vehicle, 1.5, 15, 150, and 1,500 μg/kg/day treatment groups. For basal glucose levels before HFHSD *n* = 9, 9, 8, 9, 8, and after HFHSD *n* = 10, 11, 9, 10, 10, respectively for vehicle, 1.5, 15, 150, and 1,500 μg/kg/day treatment groups. Two way ANOVA comparing within group before and after HFHSD ^*^p < 0.05, and ^**^p < 0.0125.

Mice were given a HFHSD challenge for a total of 3 days at 11 months of age (Figure 1A). Three days on the HFHSD resulted in increase in body weight and a 13–36% increase in fasting blood glucose (Figure 3). Mice were given a second metabolic challenge at 12 months of age for a total of 6 days (Figure 1B) to examine energy expenditure and activity (below). Between days 0 and 3 on the HFHSD body weight and fat mass increased 9–11% (Table 1). Between days 3 and 6 mice lost weight. This was likely due to the stress and novelty of being placed in the metabolic cages (Table 1).

**Table 1:** Body composition of female mice at 12 months of age: Estimated marginal means (± SEM)^**^ of body weight, fat mass, and lean mass.

	**Body weight (g)**			**Lean mass (g)**			**Fat mass (g)**		
Days on HFHSD	0	3	6	0	3	6	0	3	6
Vehicle	27.9 (1.1)	30.8 (1.1)	29.0 (1.2)	20.0 (0.3)	20.5 (0.3)	19.6 (0.4)	6.7 (0.7)	9.0 (0.8)	8.1 (0.9)
1.5 μg/kg/day	26.4 (1.1)	28.8 (1.1)	27.1 (1.2)	19.6 (0.3)	19.9 (0.3)	19.2 (0.3)	5.5 (0.7)	7.6 (0.8)	6.4 (0.9)
15 μg/kg/day	26.9 (1.2)	29.6 (1.2)	28.3 (1.3)	19.5 (0.3)	19.8 (0.4)	19.2 (0.4)	6.1 (0.8)	8.2 (0.9)	7.5 (1.0)
150 μg/kg/day	26.2 (0.9)	29.2 (1.0)	27.8 (1.0)	19.5 (0.3)	20.1 (0.3)	19.3 (0.3)	5.6 (0.6)	7.8 (0.7)	7.2 (0.8)
1,500 μg/kg/day	24.9 (1.0)^*^	27.8 (1.1)^*^	25.9 (1.1)^*^	19.1 (0.3)	19.6 (0.3)	18.8 (0.3)	4.8 (0.7)	6.9 (0.8)	6.1 (0.8)

*n = 10, 8, 9, 10, 9 respectively, for vehicle, 1.5, 15, 150, and 1,500 μg/kg/day treatment groups*.

Bold represents

* *p < 0.05 relative to vehicle*.

** *Models included covariates: litter, date of body weight assessment, and litter size*.

### Spontaneous Activity

At 12 months of age, mice were placed in metabolic cages on Day 4 of the HFHSD challenge. Spontaneous activity was measured over the last 48 h (Days 5–6), and each parameter was analyzed as an average of two 12 h light or dark cycles (Figure 1B). Endpoints that were measured included spontaneous activity (movement in the x, y, and z directions), ambulatory activity (movement in the x and y directions), rearing activity (z direction only), and total meters traveled (meters moved in x, y, and z directions) in the 12 h light or 12 h dark cycles).

**Figure 4:**
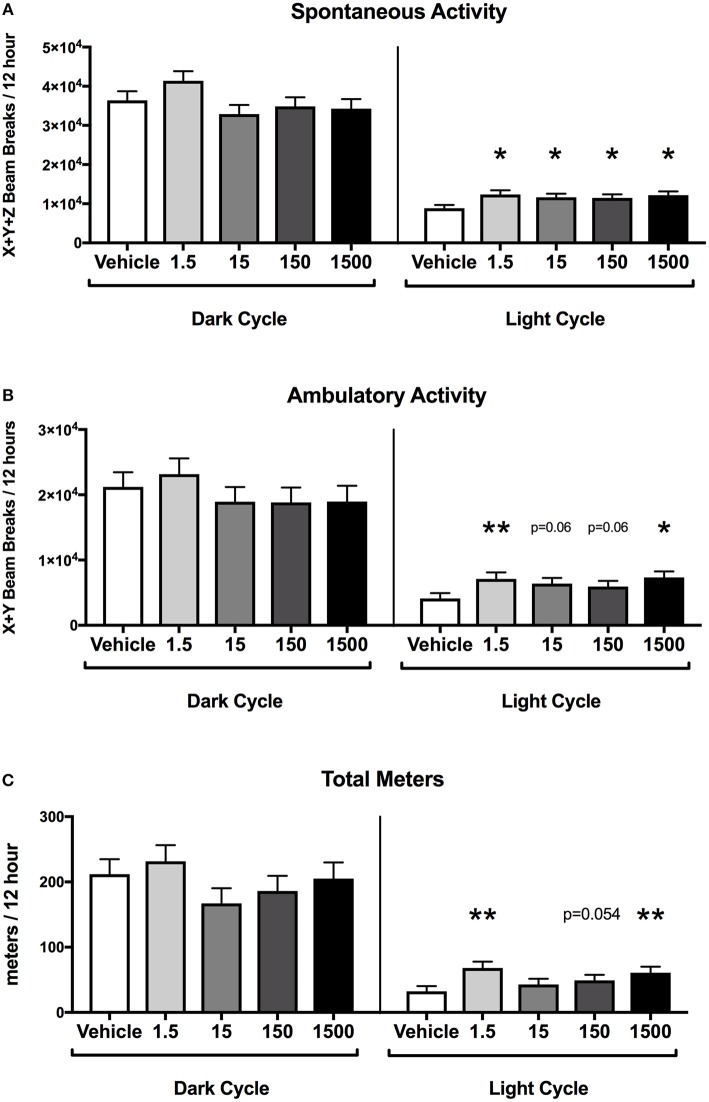
Activity of female mice at 12 months of age on a HFHSD. Estimated marginal means (±SEM) in 12 h increments of: total spontaneous activity **(A)**, ambulatory activity **(B)**, and meters traveled **(C)**. *n* = 10, 8, 9, 9, respectively for vehicle, 1.5, 15, 150, and 1,500 μg/kg/day treatment groups. ^*^p < 0.05 and ^**^p < 0.0125 relative to vehicle. Model included covariates: litter and assessment date.

Developmental exposure to UOG chemical mixture after a HFHSD metabolic challenge was associated with increased activity during the light cycle. Spontaneous activity was 30–40% and ambulatory activity was 44–78% higher in all treatment groups relative to vehicle (Figures 4A,B). Total meters traveled was 50–111% higher in the 1.5, 150, and 1500 μg/kg/day groups relative to vehicle (Figure 4C). The difference in meters traveled was not restricted to a certain time of the light cycle but was continuous throughout the light cycle (Supplementary Figure 2). Rearing activity did not differ relative to vehicle control within the light cycle (data not shown). Spontaneous activity, ambulatory activity, rearing activity, and meters traveled did not differ in treatment groups relative to vehicle during the dark cycle.

### Energy Expenditure

**Figure 5:**
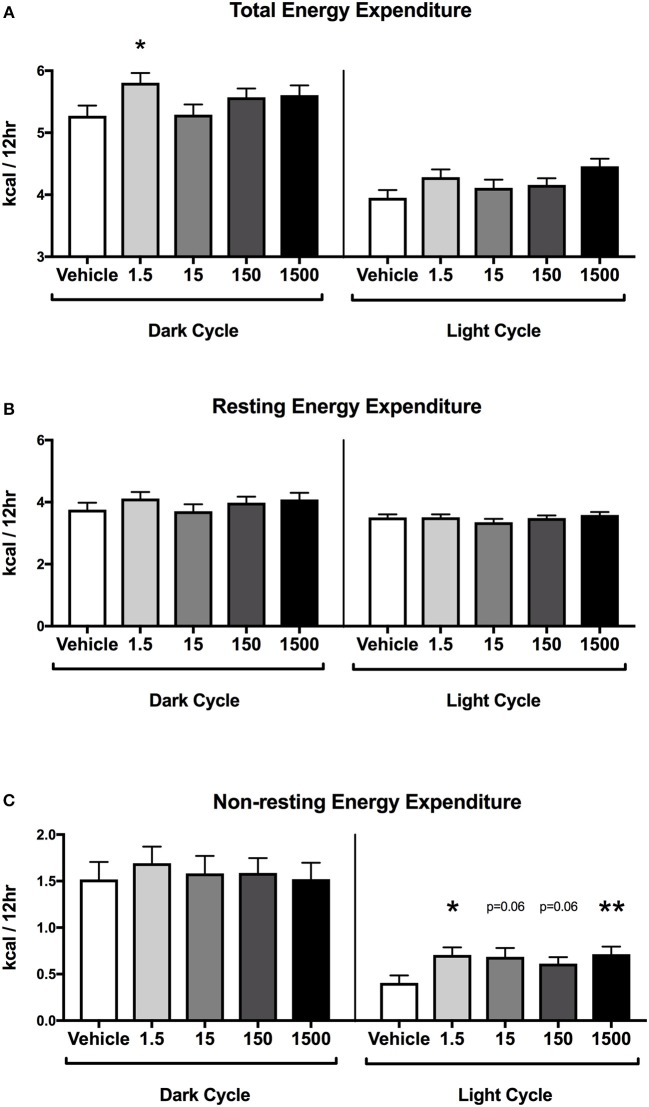
Energy expenditure in female mice at 12 months of age on HFHSD. Estimated marginal means (±SEM) in 12 h increments of: total energy expenditure **(A)**, resting energy expenditure **(B)**, non-resting expenditure **(C)**. *n* = 10, 8, 9, 9, 8, respectively for vehicle, 1.5, 15, 150, and 1,500 μg/kg/day treatment groups. ^*^p < 0.05 and ^**^p < 0.0125 relative to vehicle. Model included covariates: litter, assessment date, litter size, and body weight.

Mice were placed in metabolic cages on Day 4, which was 3 days after the HFHSD metabolic challenge began (Figure 1B). The HFHSD continued for 3 days (Days 4–6) and energy expenditure was measured over the last 48 h (Days 5–6). Each parameter was analyzed as an average of two 12 h light or dark cycles (Figure 1). Developmental exposure to UOG chemical mixture after a HFHSD metabolic challenge was associated with altered non-resting energy expenditure in the light cycle. Total energy expenditure was 13% lower in the 1,500 μg/kg/day treatment group relative to vehicle during the light cycle (Figure 5A). No differences in resting energy expenditures were observed in any of the treatment groups relative to vehicle (Figure 5B), but non-resting energy expenditure was 74, 69, 51, and 77% higher in the 1.5, 15, 150, and 1,500 μg/kg/day treatment groups, respectively, in the light cycle (Figure 5C). Total energy expenditure, resting energy expenditure, and non-resting energy expenditure did not differ in any treatment group when compared to vehicle in the dark cycle. Respiratory quotient was not different between vehicle and treatment groups in either the light or dark cycles (Supplementary Figure 3).

### Body Weight and Composition

**Figure 6:**
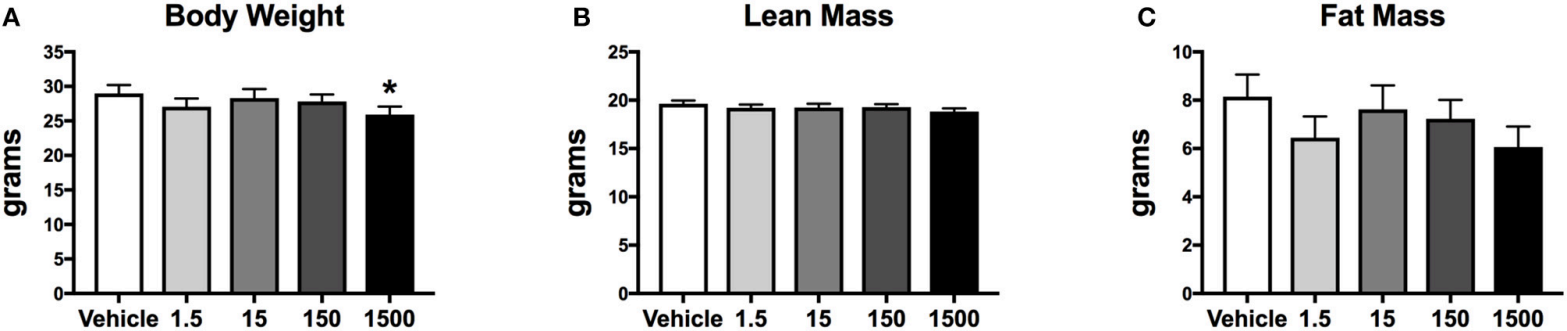
Body composition of female mice at 12 months of age after 6 days on a HFHSD after metabolic cage testing. Collapsed data from days 0, 3, and 6 of a HFHSD metabolic challenge. Estimated marginal means (±SEM) of: body weight **(A)**, nose to rump length at necropsy **(B)**, fat mass **(C)**, and lean mass. ^*^p < 0.05 relative to vehicle. *n* = 10, 8, 9, 10, 9, respectively for vehicle, 1.5, 15, 150, and 1,500 μg/kg/day treatment groups. Models included covariates: litter, date of body weight assessment, and litter size.

Developmental exposure to UOG chemical mixture altered body weight at 12 months of age. Before, during, and after the HFHSD challenge, body weight was 10% lower in the 1,500 μg/kg/day group (Figure 6A). There were no differences in fat mass or lean mass between treatment groups (Figures 6B,C). Body length (nose to rump) at necropsy was not altered in any treatment group, in comparison to vehicle (Table 1).

### Adipose Tissue and Organ Weights

**Figure 7:**
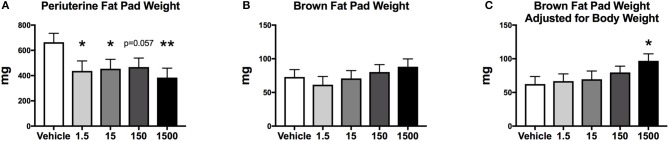
Fat pad weights of female mice at 12 months of age after HFHSD. Estimated marginal means (±SEM) of periuterine fat pad weight **(A)**, brown fat pad weight **(B)**, and brown fat weight adjusted for body weight **(C)**, ^*^p < 0.05 relative to vehicle ^**^(*n* = 10, 8, 9, 10, 9 respectively for vehicle, 1.5, 15, 150, and 1,500 μg/kg/day treatment groups). Model included covariates: litter, date.

After 6 days of the HFHSD metabolic challenge, mice were euthanized, and organs and tissues were collected and weighed. Periuterine adipose tissue weight was 35-38% less in the 1.5, 15, and 1,500 μg/kg/day groups compared to vehicle (Figure 7A). Brown adipose weight did not differ; however, the 1500 μg/kg/day group demonstrated a 55% increase in brown adipose weight compared to vehicle, when adjusted for body weight (Figures 7B,C). Kidney, heart, spleen, uterus, ovary, and liver weights, and liver triglycerides did not differ between treatment groups (Table 2).

**Table 2:** Organ and tissue weights: Estimated marginal means (±SEM) of wet organ and tissue weights.

	**Length**	**Kidney**	**Heart**	**Spleen**	**Uterus**	**Ovaries**	**Liver**	**TAG**
Vehicle	9.8 (0.1)	174.0 (4.9)	119.1 (4.3)	89.4 (6.3)	128.2 (13.2)	5.6 (0.5)	1247.5 (74.6)	23.4 (2.7)
1.5 μg/kg/day	9.8 (0.1)	177.6 (4.6)	123.6 (4.2)	90.0 (6.1)	126.1 (12.8)	5.9 (0.5)	1154.7 (81.6)	17.4 (3.0)
15 μg/kg/day	9.9 (0.1)	172.1 (5.3)	114.9 (4.6)	79.9 (6.8)	112.2 (14.2)	6.2 (0.6)	1321.8 (89.1)	17.4 (2.8)
150 μg/kg/day	9.9 (0.1)	171.6 (4.0)	118.2 (3.6)	95.3 (5.3)	129.9 (11.1)	6.1 (0.4)	1274.0 (54.9)	17.7 (2.7)
1,500 μg/kg/day	9.9 (0.1)	168.6 (4.5)	120.0 (4.1)	83.3 (5.9)	133.4 (12.5)	5.3 (0.5)	1243.4 (59.0)	19.7 (2.8)

*n = 10, 8, 9, 10, 9 respectively, for vehicle, 1.5, 15, 150, and 1500 μg/kg/day treatment groups*.

*Length = nose to rump length in cm*.

*Kidney, heart, liver, spleen, uterus, and ovary weights expressed as mg and included body weight as a covariate*.

*TAG = liver triacylglycerol mmol/kg wet weight*.

### Food Consumption and Glucose Homeostasis

Glucose tolerance tests were performed prior to initiation of the HFHSD and after 3 days on the HFHSD (Figure 1A) at 11 months of age. Serum glucose concentration did not differ before or after HFHSD metabolic challenge relative to vehicle control whether calculated at individual time points, in area under the curve, or relative to basal glucose levels (Supplementary Figure 4).

Food consumption and food bouts were measured on days 5-6 of HFHSD challenge at 12 months on age (Figure 1B). Total food consumption and food bouts did not differ between treatment groups in either the light or dark cycle (Supplementary Figure 5).

Serum insulin concentrations were measured from blood collected at necropsy, which occurred during the first 4 h of the light cycle after 6 days on the HFHSD challenge at 12 months of age. No differences were detected in insulin concentrations between vehicle and treatment groups (Supplementary Figure 6). Total islet cell number and proportion of cells types were quantified in one dose group. Pancreata were collected at necropsy, after 6 days on the HFHSD. The area of pancreas occupied by alpha, beta, and delta cells, and total islet area was quantified using immunohistochemistry did not differ between the 1,500 μg/kg/day group and vehicle (Supplementary Figure 6).

### Estrus Cyclicity

Estrus cyclicity was monitored for 2 weeks at 9.5 months of age. Overall mice tended to have irregular cycles or be in persistent estrus. Within each treatment group 90, 63, 89, 100, and 89% of mice in vehicle, 1.5, 15, 150, and 1,500 ug/kg/day, respectively, spent ≥50% of days in estrus and this did not differ relative to vehicle control. All mice appeared to be in persistent estrus at necropsy as vaginal smears contained only cornified epithelium and no white blood cells.

## Discussion

Our findings are the first to report that developmental exposure (GD1-PND21) to a mixture of 23 oil and gas chemicals increased exploratory and risk-taking activity with a subsequent increase in energy expenditure in female C57Bl/6 mice. All UOG exposure groups also had a decrease in peri-uterine fad pad weight, which may have been a consequence of increased activity and energy expenditure. Overall, treatments largely did not affect other aspects of body composition, although mice in the highest treatment group, 1,500 μg/kg/day, had some unique impacts on body composition. The HFHSD metabolic challenge caused all animals to have an increase in body weight, fat mass and higher fasting blood glucose, and did not differ between vehicle and treatment groups.

Female mice developmentally exposed to the 23 UOG mixture had an increase in exploratory activity and spontaneous activity in the light cycle. We assessed exploratory behavior using the elevated plus maze (EPM) test, and found mice developmentally exposed to the 1.5, 15, and 150 μg/kg/day UOG groups spent dramatically more time in the open arms compared to vehicle (400, 490, 290%, respectively). Since mice tend to spend more time in enclosed and protected areas for safety (29), such a robust increase in the amount of time in open arms indicates that UOG exposed mice display lower anxiety-like behavior and are more prone to risk-taking behavior in response to novel and potentially risky environment. Furthermore, when exposed to the HFHSD, these female mice that were developmentally exposed to the 23 UOG mixture, showed increased locomotor activity in the light cycle compared to vehicle mice. Mice are nocturnal and habitually rest and avoid activity in the light, a likely adaptation to evade predators. Increased activity in the light cycle could be either an indication of disturbance of circadian rhythms and sleep disorders or of hyperactive behavior. We cannot determine here if the increased light-related activity in exposed mice was already expressed as a baseline or it was induced by the diet switch or by the hypercaloric diet intake. Diets high in fat or sugar can indeed alter circadian rhythms in mammals (30). Female mice, however, have been reported to be more resistant to the disruption of daily activity rhythms during high fat feeding compared to males because of protective effects of circulating ovarian hormones (31). The 23 UOG mixture exposed female mice could thus be more sensitive to the effects of novelty or of high calories intake on activity. Taken together, increased time spent in open arms in the EPM test and increased spontaneous activity in the light cycle suggest that developmental exposure to the UOG mixture led to an enhanced response to novel stimuli (environment and food) in developmentally exposed females that may be related to high risk-taking behavior.

While determining the exact mechanisms mediating altered behavior is complex, several nuclear receptors have been shown to modulate time spent in open arms in the EPM test and the nuclear receptor antagonist activity in the UOG mixture is a likely candidate. Estrogen and androgen receptor agonists have generally been shown to decrease time spent in open arms, which is generally associated with anxiety. Postnatal exposure to DHT in Long-Evans and Wistar rats, as well as C57Bl/6 mice, resulted in less time in open arms of the EPM and less activity (32–34); this was mirrored in a joint gestational/postnatal DEHP exposure, where female offspring also spent less time in the open arms and made fewer entries into them (35). Gestational exposure to testosterone was associated with less time spent in the open arms in Wistar rats. This behavior was reversed by co-administration with the anti-androgen flutamide or the antiestrogen tamoxifen, indicating that both androgens and estrogens can regulate this behavior (36). Similarly, BPA exposure from either GD-1 to PND1 or PND1-PND8 resulted in females that spent less time in open arms in the EPM test and more anxious behavior in open field test, and a novelty test in CD-1 mice (37). As agonist activity for the androgen and estrogen receptors causes an increase in anxious behavior and a decrease in risk-taking behavior, it is possible that antagonist activity for these receptors might increase risk-taking behavior. In support of this as a possible contributory mechanism, we previously reported that 21 of the chemicals in the 23 UOG mix can antagonize the estrogen and/or androgen receptors with IC50 concentrations of approximately 1 and 5 μM, respectively (5).

We previously reported that ten chemicals in the 23 UOG mix antagonized the glucocorticoid receptor *in vitro* (5), suggesting this is a possible mechanism. Exposure to increased stress from GD3-20 in Wistar rats resulted in offspring that displayed anxious behavior: decreased time in open arms of the EPM, decreased time in the light side of the light-dark box, and decreased mobility in the forced swim test (38). However, experimentally increasing or decreasing prenatal cortisol levels exogenously with dexamethasone, betamethasone, or hydrocortisone did not lead to alterations in anxiety-like behavior in the EPM (39–41). As excess cortisol induced anxiety-like behavior in some studies, antagonistic activity could promote the opposite: an increase in risk-taking behavior.

The UOG mix also antagonizes progesterone and thyroid hormone receptors, however, these are less likely to be targets mediating the altered behavior observed in the current study. Progesterone receptor knockout mice supplemented with progesterone spent more time in the open arms of the EPM (42). Exposure to a thyroid antagonist during gestation did not impact alter time spent in open arms in the EPM test in either sex in Wistar rats (43). Given that we reported anti-thyroid and anti-progesterone activity in the UOG chemicals *in vitro*, we do not suspect these are causative mechanisms herein.

The current study involves a complex paradigm with endocrine disrupting chemical mixture exposure during development in addition to aging mice and a diet challenge, it is difficult to dissect the underlying mechanisms by which developmental UOG chemical exposure altered activity and energy expenditure in aging female mice. However, there is evidence that antagonist activity to the glucocorticoid, androgen, and estrogen receptors could be possible underlying mechanisms. These mechanisms should be substantiated through the use of targeted receptor ligand controls in future studies to assess their potential contributory roles in these effects.

While on the HFHSD, all treatment groups showed an increase in spontaneous activity and increased non-resting energy expenditure within the light cycle. Despite this increase, body weight and overall fat mass were not significantly different, though this did appear to contribute to a significant reduction in peri-uterine fat pad mass in the developmentally exposed animals. However, a limitation of the metabolic cages is that energy expenditure is only taken at one time point and cannot reflect the metabolism of an animal throughout its lifespan. While mice were allowed 24 h to acclimate to the cages, they did lose weight across the 3 days spent in the metabolic cages, so it is possible that stress is a complicating factor in the results of the current study.

In the current study, four different doses of the same treatment mixture spanned three orders of magnitude. Doses were selected to include environmentally relevant concentrations to mimic chemical concentrations found to be reported in UOG drilling regions. The concentrations in the drinking water of the 1,500 μg/kg/day group mimic those reported in industry wastewater samples, the 150 μg/kg/day group mimic those reported in surface and groundwater at sites of UOG fluid spills, and the 1.5 and 15 μg/kg/day groups mimic concentrations reported in surface and groundwater UOG regions without reported spills (44–46). The lower dose groups are potentially within the range of current human exposures for those living in UOG drilling dense areas.

Given the broad dose response and the known EDC antagonist activity of the UOG mixes, we anticipated and found quantitatively and qualitatively different effects at low vs. high doses across the broad dose response following developmental exposure (47). For example, the 1500 μg/kg/day UOG chemical dose group was unique. These animals exhibited a significant decrease in body weight. Energy expenditure and activity were similar to other dose groups that did not share this body weight difference, though these animals did also have a significant increase in brown adipose, which may have impacted metabolic health. The 1,500 μg/kg/day group also did not show an increase in time spent in open arms in the EPM test as observed in other treatment groups, suggesting differing mechanisms promoting these effects. Future studies directed at this treatment group should include looking further into thermogenic factors of brown adipose, for example, expression of Prdm16, UCP-1 or PGC-1α.

Many developmental programming phenotypes need a secondary adult challenge to be revealed. At seven months of age, these mice exhibited decreased resting energy expenditure and activity (17). To investigate whether aging and a metabolic challenge would exacerbate this phenotype, these mice were aged to 12 months and given a HFHS diet challenge (current study). This led to an overall increase in energy expenditure and activity in treatment groups compared to vehicle. While we did not see an exacerbation of the effects seen at 7 months, age plus a HFHS diet was associated with increased spontaneous activity in treatment groups in the light cycle. Both age and the HFHSD can alter behavior and metabolism and it is possible that the combination could result in unanticipated opposing actions. Future studies are needed to separately examine these two variables to completely understand how age and diet interact with developmental exposure to the UOG mix to modulate activity and energy expenditure in adulthood.

Further research is needed to more conclusively determine the mechanisms driving these effects and substantiate these findings as the current study has limitations. The dams utilized herein were exposed twice to the same concentration of UOG mix for two sets of experiments during pregnancy; we cannot rule out that this double exposure in part contributed to the observed effects. While further research utilizing a single exposure should be performed to substantiate these findings, the current experimental paradigm is completely relevant to women who have more than one child while living in UOG areas. We also evaluated only female mice, and further investigation of males should be explored in future work to assess impacts on male offspring and potential sex differences in these effects. We have previously reported that a prenatal only exposure to the highest doses altered testosterone concentrations in adult males (5); it also altered some pituitary hormones, but did not alter estradiol concentrations in adult females (19). Future studies should evaluate these hormones and others, like cortisol, in F0 and F1 mice. Lastly, we assessed potential effects after a short diet challenge, which has been previously shown to disrupt metabolic health; however, chronic exposure to a HFHSD would more closely mirror a western diet.

Taken together, developmental exposure to the 23 UOG mixture was associated with increased activity and non-resting energy expenditure in the light cycle, increased exploratory behavior in the EPM test, and decreased sleep and fat pad weight in 12 month female mice. All of these effects were seen in the light cycle when mice are normally less active, suggesting potential adverse effects. Increased risk-taking behavior and decreased sleep could be factors associated with attention deficit and hyperactivity disorders, along with major depressive disorders (48). Interestingly in humans, an association between living close to an unconventional natural gas development and symptoms of depression has been reported (49). Further studies are needed to better understand the behavioral changes observed after developmental exposure to UOG chemicals.

## Ethics Statement

This study was carried out in accordance with the recommendations National Research Council's Guide for the Care and Use of Laboratory Animals. The protocol was approved by the University of Missouri Animal Care and Use Committee.

## Author Contributions

VB and JC-G performed animal experiments. VB analyzed the data. DR and RR provided pancreas analysis. All authors have contributed to the design of experiments and interpretation of the data.

### Conflict of Interest Statement

The authors declare that the research was conducted in the absence of any commercial or financial relationships that could be construed as a potential conflict of interest.
