# Supplementary material for: Developmental Exposure to a Mixture of Unconventional Oil and Gas Chemicals Increased Risk-Taking Behavior, Activity and Energy Expenditure in Aged Female Mice After a Metabolic Challenge
Source: Front Endocrinol (Lausanne). 2019 Jul 25;10:460. doi: 10.3389/fendo.2019.00460 (PMC6669236; doi:10.3389/fendo.2019.00460)
Supplement: Supplementary file 1 [file Data_Sheet_1.docx]

Supplementary Figure 1. Maternal Health. *Reported in Balise et al 2019 [1].*Health of dams shown through estimated marginal means (+/-) SEM of body weight at gestational day zero before exposure (A), water consumption throughout exposure (B), dams that were shown to be plug positive and deliver (C), and total number of pups at delivery (D) (n= 14, 9, 11, 8, 10 respectively for vehicle, 1.5, 15, 150, and 1500 µg/kg/day treatment groups).

Supplementary Table 1. Sample size for dams, F1 pups, and unique litters.

|  | Dose (µg/kg/day) | | | | |
| --- | --- | --- | --- | --- | --- |
|  | Vehicle | 1.5 | 15 | 150 | 1500 |
| Dams that were mated | 14 | 9 | 11 | 8 | 10 |
| Dams that plugged | 14 | 9 | 11 | 8 | 10 |
| Dams that delivered | 11 | 6 | 9 | 5 | 6 |
| Cannibalized | 3 | 1 | 0 | 0 | 1 |
| Dams with pups at PND7 | 8 | 5 | 9 | 5 | 5 |
| litter used in separate study | 0 | 0 | 3 | 0 | 0 |
| Excluded at least 1 male or female, litter>3 | 1 | 1 | 1 | 1 | 1 |
| Individual Litters left in study | 6 | 4 | 5 | 4 | 4 |
| Animals in Body Composition | 10 | 8 | 9 | 10 | 9 |
| Distribution of number of animals in individual litters | 2, 1, 1, 3, 1, 2 | 2, 4, 1, 1 | 2, 3, 1, 2, 1 | 1, 4, 1, 4 | 2, 1, 2, 4 |
| Subset with valid Indirect Calorimetry | 10 | 7 | 9 | 10 | 8 |
| Indirect Calorimetry Distribution of Number of Animals in Individual Litters | 2, 1, 1, 3, 1, 2 | 2, 3, 1, 1 | 2, 3, 1, 2, 1 | 1, 4, 1, 4 | 1, 1, 2, 4 |
| Respective Litter Size of Above Litters | 6, 5, 7, 4, 4, 5 | 9, 7, 4, 6 | 7, 9, 7, 9, 7 | 4, 7, 5, 7 | 5, 4, 7, 6 |

Supplementary Figure 2. Meters Traveled over 48 hours. Average meters travelled over 48 hour timespan (6am-6am) n=10, 8, 9, 9, respectively for vehicle, 1.5, 15, 150, and 1500 µg/kg/day treatment groups. Light cycle(6am-6pm), dark cycle represented by grey rectangle (6pm-6am).


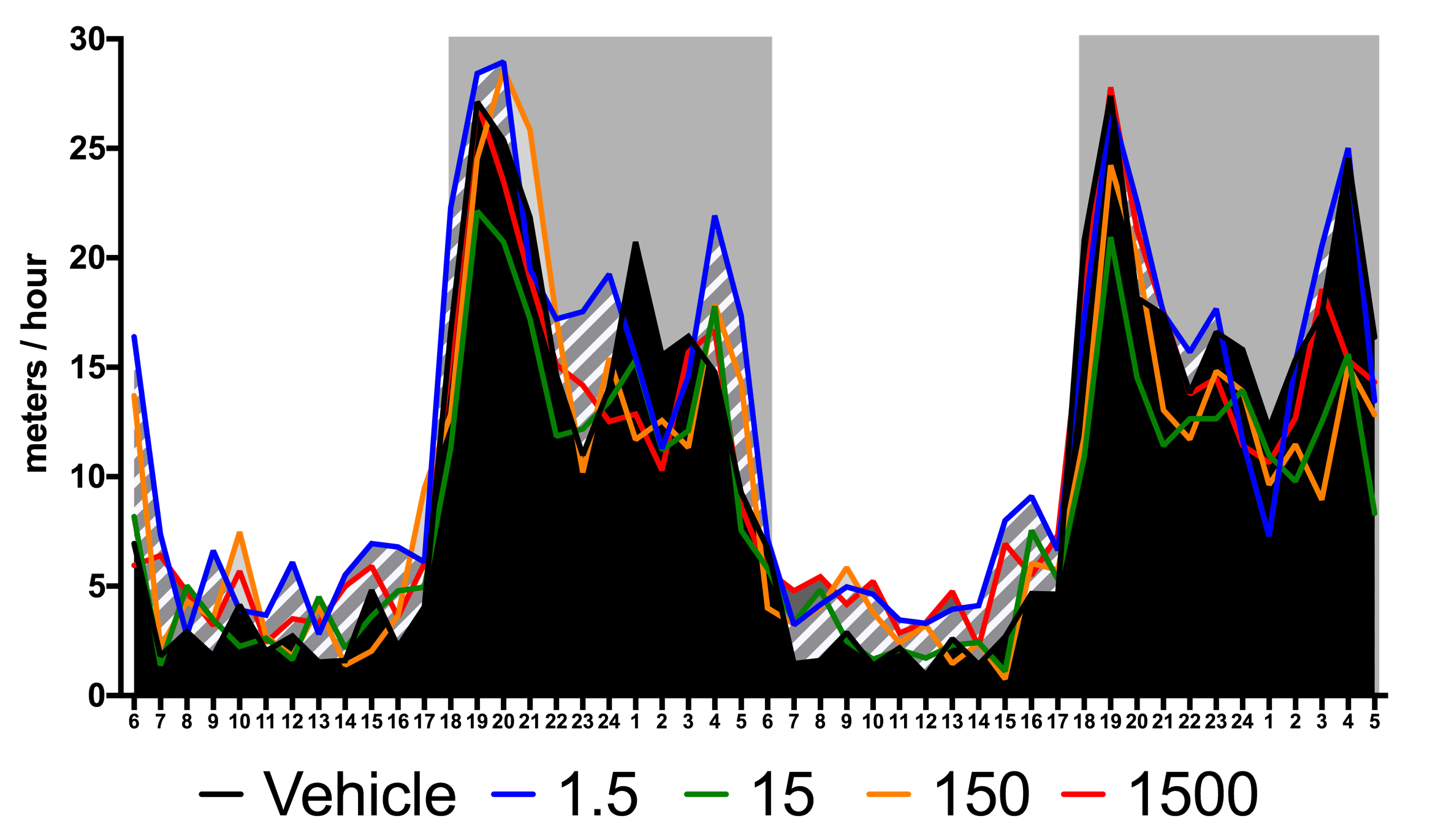


Supplementary Figure 3. Respiratory quotient of female mice at 12 months of age on a HFHSD. Estimated marginal means (+/- SEM) in 12-hour increments of respiratory quotient. n=10, 8, 9, 9, respectively for vehicle, 1.5, 15, 150, and 1500 µg/kg/day treatment groups. Model included covariates: litter and assessment date.


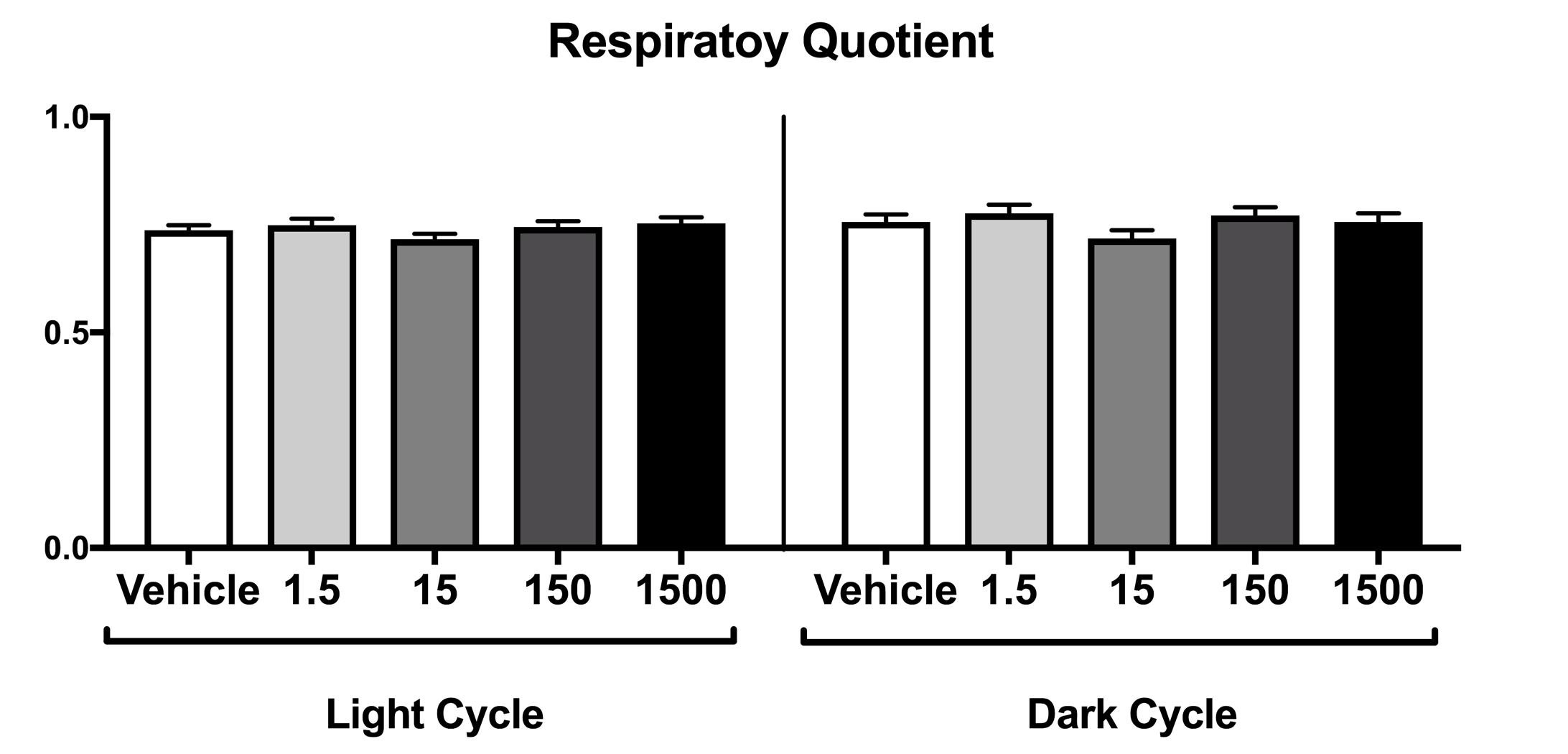


**Supplementary Figure 4. Glucose tolerance test of female mice at 10 months of age.** Estimated marginal means (+/- SEM) of blood glucose levels at time points 0, 30, 60, and 120 minutes after glucose injection (A), blood glucose levels at time points 0, 30, 60, and 120 minutes after glucose injection after a HFHSD (B), area under the curve of glucose tolerance test for before and after HFHSD (C). (Before HFHSD n= 9, 9, 8, 9, 8, After HFHSD n= 10, 11, 9, 10, 10 respectively for vehicle, 1.5, 15, 150, and 1500 µg/kg/day treatment groups. * p<0.05, and ** p<0.0125 relative to vehicle. Model included litter as a covariate.)

Supplementary Figure 5. Food Consumption of Female Offspring at 12 months of age. Estimated marginal means (+/-) SEM for total food consumption in dark and light cycle (A). (n= 7, 8, 9, 10, 5 respectively for vehicle, 1.5, 15, 150, and 1500 µg/kg/day treatment groups. Model included covariates: litter and assessment date.)

Supplementary Figure 6. Pancreatic cell quantification and serum insulin concentration in female mice at 12 months of age after HFHSD. Estimated marginal means (+/- SEM) of pancreatic cells alpha, beta, and delta compared to the total islet area after HFHSD (A), serum insulin levels at necropsy after HFHSD (B). (n=7,7 respectively for vehicle and 1500 µg/kg/day treatment groups. * p<0.05, and** p<0.0125 relative to vehicle. Model included litter as a covariate.)


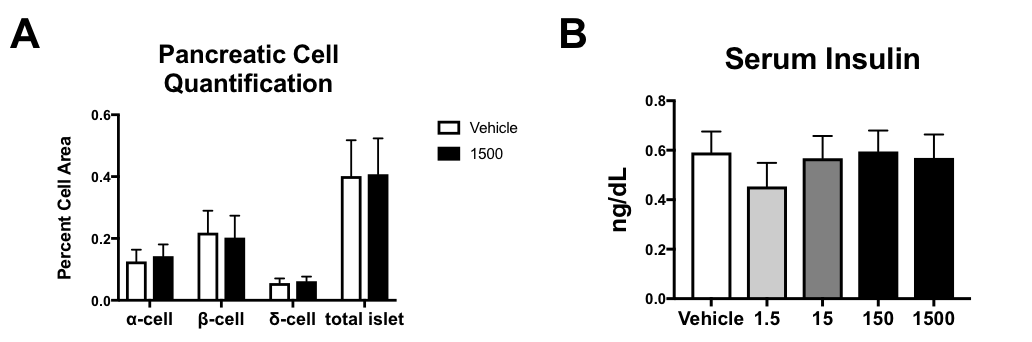


[1] V.D. Balise, J.N. Cornelius-Green, C.D. Kassotis, R.S. Rector, J.P. Thyfault, and S.C. Nagel, Preconceptional, Gestational, and Lactational Exposure to an Unconventional Oil and Gas Chemical Mixture Alters Energy Expenditure in Adult Female Mice. Front Endocrinol (Lausanne) 10 (2019) 323.
